# Phase II assessment of talabostat and cisplatin in second-line stage IV melanoma

**DOI:** 10.1186/1471-2407-9-263

**Published:** 2009-07-30

**Authors:** Robert M Eager, C Casey Cunningham, Neil N Senzer, Joe Stephenson, Stephen P Anthony, Steven J O'Day, Gary Frenette, Anna C Pavlick, Barry Jones, Margaret Uprichard, John Nemunaitis

**Affiliations:** 1 Mary Crowley Cancer Research Centers, Dallas, TX, USA; 2 Texas Oncology PA, Dallas, TX, USA; 3 Baylor Sammons Cancer Center, Dallas, TX, USA; 4 Gradalis, Inc., Dallas, TX, USA; 5 Clinical Research Unit, Cancer Centers of the Carolinas, Greenville, SC, USA; 6 Cancer Care Northwest, Spokane, WA, USA; 7 The Angeles Clinic and Research Institute, Los Angeles, CA, USA; 8 Carolinas Medical Center, Charlotte, NC, USA; 9 Department of Dermatology, New York University Clinical Cancer Center, New York, NY, USA; 10 Point Therapeutics, Inc., Boston, MA, USA; 11 Department of Internal Medicine, The University of Hawaii, Honolulu, HI, USA

## Abstract

**Background:**

Metastatic melanoma is an incurable disease with an average survival of less than one year. Talabostat is a novel dipeptidyl peptidase inhibitor with immunostimulatory properties.

**Methods:**

This phase II, open label, single arm study was conducted to evaluate the safety and efficacy of 75–100 mg/m^2 ^cisplatin combined with 300–400 mcg talabostat bid for 6, 21-day cycles. The primary endpoint was overall response. The rate of complete responses, duration of overall objective response, progression-free survival (PFS), and overall survival were the secondary endpoints.

**Results:**

Six objective partial responses were recorded in the 74 patients (8.1%) in the intention-to-treat population. Five of these responses involved the 40 evaluable patients (12.5%). Thirty-one percent of patients reported SAEs to the combination of talabostat and cisplatin.

**Conclusion:**

Acceptable tolerability was observed in the intention-to-treat population and antitumor activity was observed in 12.5% of evaluable patients, which is not greater than historical expectation with cisplatin alone.

## Background

Therapeutic options for advanced melanoma are limited to palliative management. No treatments have demonstrated survival advantage once metastatic lesions develop. Combination therapy produces the highest response rates, but overall survival remains less than 12 months. The combination chemotherapy of cisplatin/vinblastine/dacarbazine produces a response rate of 40% and a median overall survival of 9 months [1]. The regimen of cisplatin/dacarbazine/carmustine with or without tamoxifen results in a 15–52% response rate and median survival of 6.8–10.8 months [2-4]. The addition of the biochemotherapeutic agents, interferon-alpha (IFN-α) or interleukin-2 (IL-2), separately or in combination, produces a modest improvement in response rate, but without survival benefit [5-13] and with increased toxicity. Single-agent treatment has similarly failed to significantly improve survival; agents that have been used include dacarbazine [14], temozolomide [15], cisplatin [16], and nitrosoureas [15,17].

Talabostat (valine-proline-boronic acid) together with other amino boronic dipeptides was originally designed as a high affinity, competitive inhibitor of the enzyme dipeptidyl peptidase IV (DPP-IV or CD26) [18]. The compound was found to stimulate hematopoiesis and antitumor immune responses via cytokine upregulation [19-21]. In addition to DPP-IV, the dipeptidyl peptidases 8 and 9 (DPP-8 and DPP-9) and fibroblast activation protein (FAP) were subsequently shown to be sensitive to inhibition by talabostat [22,23]. Based on similarities of protein structure and substrate specificity, DPPs-8 and -9 and FAP are classified as members of the DPP-IV-like family of post-prolyl cleaving serine proteases [24].

DPPs-8 and -9 are cytosolic proteases and their inhibition by talabostat has been shown to cause caspase-1 activation and IL-1β induction in macrophages, which in turn causes upregulation of the cytokines and chemokines that characterize the responses to talabostat, both *in vitro *and in tumor-bearing mice [23]. FAP has previously been described as a type II membrane protein with dipeptidyl peptidase and gelatinase activity (reviewed in [24]). Studies of FAP have reported that FAP expression is induced in fibroblasts associated with the stroma of malignant epithelial tumors and healing wounds [25-27]. These reports suggest that FAP does not appear to be expressed constitutively in most healthy tissues of the adult animal; although FAP expression in bone marrow and lymphoid tissue from both healthy and tumor-bearing mice has been demonstrated [22]. FAP, therefore, represents a molecular target for talabostat in tumor stroma; but the involvement of FAP in the antitumor effects of talabostat in mouse tumor models is currently unclear.

The biological activities of the cytokines and chemokines upregulated by talabostat suggest that both innate and adaptive immunity are evoked. In animal models, talabostat enhanced the production of cytokines in tumor tissue and lymphoid organs, resulting in enhanced tumor-specific T-cell-dependent [19] and T-cell-independent [20,21] immunity. These antitumor responses were enhanced by concomitant treatment with chemotherapeutic agents, including cisplatin, gemcitabine, paclitaxel, 5-fluorouracil, and the monoclonal antibody rituximab [21]. The mechanism underlying the synergistic effects is unknown; but it may involve the exposure of tumor antigens by chemotherapy-induced cellular apoptosis in tumor models in immunocompetent mice [28].

Clinical studies have shown talabostat to be well tolerated [29-31]. In a phase I trial in thirteen patients treated concomitantly with immunosuppressive chemotherapy, five patients showed improvement in grade 3 neutropenia and most developed elevations in serum cytokine levels [29]. A phase I trial of talabostat and rituximab in rituximab-resistant lymphoma showed cytokine elevations in most patients with partial response in 3 patients [31]. However, a phase II trial of talabostat in metastatic colorectal carcinoma reported no objective responses [32].

The cooperative or synergistic interactions observed with cisplatin and dacarbazine are particularly relevant to the investigation of the activity of talabostat in metastatic melanoma. In mouse models of WEHI 164 and EL4 tumors, the combination treatment of established tumors with talabostat and cisplatin revealed a significantly enhanced antitumor effect compared with either agent alone [20]. Mice rendered tumor-free following treatment with talabostat and cisplatin were resistant to rechallenge with tumors cells of the primary type, suggesting the development of therapy-based immune protection. Based on these findings, we investigated the effects of talabostat in combination with cisplatin in patients with metastatic melanoma.

## Methods

### Patient Population

Patients were eligible for inclusion if they were 18 years or older with histologically or cytologically confirmed metastatic melanoma, Stage IV according to the AJCC [33], with measurable disease per Response Evaluation Criteria in Solid Tumors (RECIST) with at least one measurable index lesion with clearly defined margins documented by spiral computed tomography (CT) or magnetic resonance imaging (MRI). Color medical photograph for skin and oral lesions or plain X-ray also were done to evaluate presence of disease. A radiated lesion was be considered an index lesion unless there was evidence of disease progression at that site prior to first administration of study medication. Additional inclusion criteria were Eastern Cooperative Oncology Group (ECOG) performance status of 0, 1, or 2; and an expected survival of ≥ 12 weeks. Patients with previously irradiated and/or resected asymptomatic central nervous system (CNS) metastases were eligible for the study.

Patients were excluded from study participation if they had received more than one prior chemotherapy or immunotherapy regimen for Stage IV melanoma, or radiation therapy to >50% of the pelvis. Patients were also excluded from the study if they had clinically significant abnormalities in laboratory tests for hepatic, biliary, renal or hematological function, per normal laboratory parameters. Additional factors disqualifying a potential patient included the following: symptomatic CNS metastases with or without significant edema; <3 weeks since prior focused radiotherapy for brain metastases or <4 weeks since prior whole brain radiotherapy; the need for chronic (i.e., >7 days) oral or intravenous (IV) corticosteroid therapy with >10 mg/day prednisone equivalents; any comorbidity or condition which, in the opinion of the investigator, interfered with the assessments and procedures of the protocol; or any malignancy within the 5 years immediately prior to the first dose of study medication with the exception of basal cell or non-metastatic squamous cell carcinoma of the skin and carcinoma in situ of the cervix. Patients were also excluded if they were within 30 days of surgery, chemotherapy, radiation therapy, immunotherapy, or other investigational medication for melanoma; patients must have recovered from all of the side effects of treatment in order to be enrolled. Women were excluded if they were pregnant or lactating. Women of childbearing potential and non-vasectomized men agreed to use a barrier method of contraception during treatment.

### Study Design

This was a Phase II open-label, single-arm, multicenter study designed to enroll up to 54 evaluable patients, with 19 evaluable patients to be enrolled into Stage 1 (safety assessment) of the study. Upon satisfactory evaluation of safety and initial evidence of antitumor activity, an additional 35 evaluable patients were enrolled. Patients received combination therapy with cisplatin and Talabostat.

The dose of cisplatin was 100 mg/m^2 ^administered intravenously on Day 1 of each 21-day cycle for 6 cycles.

Talabostat 300 mcg was administered orally twice daily (BID) from Days 2 through 15 of Cycle 1. In Cycle 2 or subsequent cycles, the talabostat dose could be increased to 400 mcg BID depending on tolerability. Patients received study treatment in 21-day cycles for up to 6 cycles. If cisplatin was discontinued for toxicity talabostat could be continued for subsequent cycles as single agent.

All patients were to be followed for progressive disease (PD) and/or survival for up to 12 months following their last dose of talabostat, unless another anti-melanoma treatment was initiated.

The study was conducted in accordance with current US Food and Drug Administration (FDA) regulations, good clinical practice (GCP), the International Conference on Harmonisation (ICH) guidelines, the version of the Declaration of Helsinki current at the time of the study, and local ethical and legal requirements. Informed consent was signed by each patient prior to his or her participation in the study.

### Study Objectives

The primary objective was to determine objective response rate, defined as the proportion of evaluable patients who achieved a complete response (CR) or partial response (PR) per RECIST. Objective response was determined using an imaging technique (e.g. CT, MRI) including all index lesions. Confirmation of response was to be documented by repeat imaging no sooner than 4 weeks following documentation of the initial response. Patients who did not qualify for a CR or PR were evaluated as having either stable disease (SD) or progressive disease (PD).

The rate of CR, duration of overall objective response, progression-free survival (PFS), and overall survival were the secondary measures of antitumor activity. Duration of response was defined as the time interval measured in days between the first date on which the criteria for objective response were met and the first date on which objective progression was documented. PFS was defined as the interval between the date of first study treatment and the first date (plus one day) on which criteria for PD or death were met.

Safety assessments were also a secondary objective. Occurrence and severity of AEs, physical examinations, weight, vital signs, ECOG performance scores, clinical laboratories (hematology, chemistry, and coagulation), and urinalysis were collected. ECGs were recorded at baseline, end of treatment and at the 30-day follow-up visit.

### Statistical Analysis

Continuous variables were summarized descriptively using mean, standard deviation (SD), median, minimum (min), and maximum (max). Categorical variables were summarized descriptively through the use of number and percent. All statistical analyses were carried out using SAS^® ^version 8.2 running on Windows XP.

The evaluable population (N = 40) was defined as those patients who received at least 21 days of talabostat (i.e., 75% of the planned doses) in Cycles 1 and 2, had no more than 7 days of vomiting in the first two cycles, and had a post-baseline response assessment. The intention-to-treat (ITT) population (N = 74) was defined as all patients who received at least one dose of talabostat. The safety population (N = 74) was defined as those patients who received any dose of talabostat or cisplatin. In this study, these 2 populations were the same. The ITT population was used in analyses of the secondary endpoints.

## Results

### Patient Characteristics

Initially, up to 54 evaluable patients were to be enrolled in this study, with enrollment proceeding in 2 stages. Stage 1 was to be completed after 19 evaluable patients were enrolled.

However, initial evaluation of safety revealed a high unevaluability rate related to the higher dose of cisplatin. This cisplatin dose was reduced from 100 mg/m^2 ^to 75 mg/m^2 ^and additional patients were placed on trial for a total of 74 patients. Forty were evaluable for response (defined as those patients who received at least 21 days of talabostat in Cycles 1 and 2). In both the ITT and Evaluable populations, the median age was 58.0 years (range, 27–79). In the ITT population, nearly all patients were white, non-Hispanic (69/74 [93.2%]). Fifty patients (67.6%) were male. Additional demographic and baseline clinical data is presented in Table 1.

**Table 1:** Baseline demographic data and disease characteristics of enrolled patients.

	ITT Population (N = 74)	Evaluable Population (N = 40)
**Age, yrs**		
Median (range)	58.0 (27–79)	58.0 (32–79)
Mean (SD)	57.6 (12.79)	58.9 (13.08)
**Race, n (%)**		
White, Non-Hispanic	69 (93.2)	39 (97.5)
Black, Non-Hispanic	2 (2.7)	1 (2.5)
Hispanic	2 (2.7)	0
Asian or Pacific Islander	1 (1.4)	0
**Gender, n (%)**		
Male	50 (67.6)	28 (70.0)
Female	24 (32.4)	12 (30.0)
**Tobacco use, n (%)**		
Never	32 (43.2)	14 (35.0)
Past	26 (35.1)	17 (42.5)
Current	16 (21.6)	9 (22.5)
**ECOG performance status, n (%)**		
0	32 (43.2)	20 (50.0)
1	42 (56.8)	20 (50.0)
**Time since initial melanoma diagnosis, months**		
Median (range)	28.0 (1–420)	30.5 (3–225)
Mean (SD)	50.1 (66.28)	45.5 (45.43)
**Time since first diagnosis of Stage IV melanoma, months**		
Median (range)	4.0 (0–89)	5.0 (0–30)
Mean (SD)	9.3 (15.66)	7.5 (7.61)
**Stage at original diagnosis, n (%)**		
Stage 0	1 (1.4)	0
Stage I	10 (13.5)	3 (7.5)
Stage II	16 (21.6)	8 (20.0)
Stage III	26 (35.1)	15 (37.5)
Stage IV	18 (24.3)	11 (27.5)
Stage unknown	3 (4.1)	3 (7.5)
**Histologic subtype, n (%)**		
Superficial spreading melanoma	17 (23.0)	7 (17.5)
Nodular melanoma	30 (40.5)	20 (50.0)
Acral lentiginous melanoma	3 (4.1)	0
Other	24 (32.4)	13 (32.5)
**Current M classification by sites of metastases^a^, n (%)**		
M1a: Distant skin, subcutaneous, or nodal metastases	7 (9.5)	4 (10.0)
M1b: Lung metastases	13 (17.6)	7 (17.5)
M1c: All other visceral metastases	54 (73.0)	29 (72.5)

ITT = intention-to-treat; SD = standard deviation; ECOG = Eastern Cooperative Oncology Group

**a **Per 2002 AJCC. Baseline LDH levels were used in this classification.

### Efficacy

Six objective responses, all PRs, were recorded in the 74 patients in the ITT Population for a response rate of 8.1% (6/74). Five of these responses were in the evaluable population, for a response rate of 12.5% (5/40). Response assessments based on RECIST criteria and are summarized in Table 2.

**Table 2:** Overall objective response rate of patients at cycle 3 and maintained to cycle 5 or end of treatment.

Timepoint	ITT Population	Evaluable Population
Overall response to treatment^a^	(N = 74)	(N = 40)
**Cycle 3, Day 1**		
CR	0	0
PR	3 (4.1)^b^	3 (7.5)
SD	34 (45.9)	25 (62.5)
		
**Cycle 5, Day 1**		
CR	0	0
PR	3 (4.1)	3 (7.5)
SD	16 (21.6)	13 (32.5)
		
**End of treatment or early termination**		
CR	0	0
PR	2 (2.7)	1 (2.5)
SD	10 (13.5)	7 (17.5)

ITT = intention-to-treat; CR = complete response; PR = partial response; SD = stable disease; PD = progressive disease

**a **Response is based on the investigator's assessment using RECIST.

**b **N (%).

The most common assessment of SD was documented in 25/40 (62.5%) of evaluable patients at cycle 3, day 1 and 13/40 (32.5%) at Cycle 5, Day 1. The duration of stable disease in 12 evaluable patients was not maintained out to cycle 5. For the patients who responded, the duration of the response ranged from 62 to 287 days. Table 3 presents disease assessments, prior treatments, and the response to talabostat for all patients with objective overall responses.

**Table 3:** Objective response to talabostat.

Age/Sex (Patient No.)	M classification at enrollment^a^	Metastatic sites at enrollment	Prior Tx for Stage IV disease^b^	Best response to prior Tx	Response to talabostat	Duration of Response (days)
54/F (01-003)	M1b	Skin, lung	Tumor peptide heat shock (Vitespen)	PD	PR	151
46/M (04-002)	M1b	Lung	Sargramostim	PD	PR	62
63/M (04-003)	M1b	Lung	None	NA	PR	287
61/F (06-001)	M1c	Subcutaneous tissue, stomach, lymph node(s), visceral	Temozolomide, thalidomide	SD	PR	Unknown^c^
53/M (09-014)	M1c	Regional lymph node(s), liver	None	NA	PR	176
58/F (12-003)	M1c	Lymph nodes beyond regional, lung, abdominal wall	None	NA	PR	141

Tx = treatment; PD = progressive disease; PR = partial response; NA = not applicable; SD = stable disease

**a **M1a: Distant skin, subcutaneous, or nodal metastases; M1b: Lung metastases; M1c: all other visceral metastases. Baseline LDH levels were also used in this classification.

**b **Includes chemotherapy and/or immunotherapy.

**c **The patient was lost to follow-up.

The estimate of median PFS using the Kaplan-Meier survival analysis algorithm for the ITT population was 92.0 days based on the investigator assessment using RECIST. Table 4 presents the overall median PFS estimate. The estimates of PFS was suggested to be greater for patients with a M1b classification and escalated dose of talabostat.

**Table 4:** Progression free survival

	----------Time to PD or Death (days)^a^----------
	Median (95% CI), days
**Overall PFS (N = 74)^b^**	92.0 (79.0, 126.0)

**Exploratory Analyses**	
	
**PFS by:**	
**M classification**	
M1a (n = 7)	85.0 (40.0, 270.0)
M1b (n = 13)	135.0 (103.0, 205.0)
M1c (n = 54)	85.0 (68.0, 110.0)
**Prior chemotherapy**	
With (n = 40)	89.0 (78.0, 143.0)
Without (n = 34)	97.0 (42.0, 180.0)
**Dose escalation of talabostat**	
With (n = 44)	135.0 (89.0, 170.0)
Without (n = 30)	79.0 (42.0, 109.0)
**Initial cisplatin dose**	
100 mg/m^2 ^(n = 35)	80.0 (44.0, 103.0)
75 mg/m^2 ^(n = 39)	126.0 (85.0, 169.0)

PD = progressive disease; PFS = progression-free survival; CI = confidence interval

**a **Response is based on the investigator's assessment.

**b **ITT population.

For the ITT Population, the estimate of median overall survival using the Kaplan-Meier algorithm was 230.0 days, regardless of whether patients starting a new anti-melanoma therapy were censored. Table 5 presents median survival estimates for the ITT Population overall, and by M classification, prior chemotherapy treatment, dose escalation with talabostat, or initial cisplatin dose. The overall survival estimates were greater for patients who did not receive prior chemotherapy (340.0 vs. 165.0 days) and for those who dose-escalated with talabostat (330.0 vs. 139.0 days). The initial cisplatin dose level did not significantly impact overall survival.

**Table 5:** Overall survival

	------------------------------Time to Death (days)------------------------------	
	Analysis with censoring of patients who began a new therapy^a^	Analysis without censoring of patients who began a new therapy^b^
	Median (95% CI), days^c^	Median (95% CI), days^c^
**Overall Survival (N = 74)**	230.0 (143.0, 401.0)	230.0 (148.0, 330.0)

**Exploratory Analyses**		
		
**Overall survival by:**		
**M classification**		
M1a (n = 7)	NE (270.0, NE)	340.0 (270.0, NE)
M1b (n = 13)	NE (230.0, NE)	NE
M1c (n = 54)	148.0 (116.0, 297.0)	148.0 (117.0, 239.0)
**Prior chemotherapy**		
With (n = 40)	165.0 (118.0, 297.0)	165.0 (143.0, 297.0)
Without (n = 34)	401.0 (138.0, NE)	340.0 (139.0, NE)
**Dose escalation of talabostat**		
With (n = 44)	330.0 (153.0, NE)	330.0 (165.0, NE)
Without (n = 30)	139.0 (81.0, 270.0)	139.0 (81.0, 230.0)
**Initial cisplatin dose**		
100 mg/m^2 ^(n = 35)	230.0 (109.0, NE)	239.0 (116.0, 401.0)
75 mg/m^2 ^(n = 39)	209.0 (148.0, NE)	209.0 (148.0, 340.0)

CI = confidence interval; NE = non-estimable

**a **Patients not dying had their survival time censored on the last date of known contact. Patients who began another anti-melanoma therapy were censored on the start date of the new anti-melanoma treatment.

**b **Patients not dying had their survival time censored on the last date of known contact. Patients who began another anti-melanoma therapy were not censored because of the new therapy.

**c **In many cases, there was insufficient information to calculate the median or the upper limit for the CI for these data.

**d **ITT population.

### Safety

All enrolled patients who received any talabostat or cisplatin were included in the safety population (N = 74). The majority of adverse events (AEs) (821/1071 [76.7%]) occurred during the first 3 cycles of treatment. Forty-two patients (42/74 [56.8%]) experienced grade 3 or grade 4 AEs, 23/74 (31.1%) patients reported SAEs, and 14/74 (18.9%) patients discontinued talabostat due to an AE. A summary of AEs by cycle and overall is presented in Table 6. AEs experienced by ≥ 3 patients (4.1%) in the Safety Population are listed in Table 7 by system organ class, with the preferred terms listed in decreasing frequency.

**Table 6:** Adverse events.

	Cycle 1 (n = 74)^a^	Cycle 2 (n = 61)	Cycle 3 (n = 38)	Cycle 4 (n = 32)	Cycle 5 (n = 19)	Cycle 6 (n = 18)	>Cycle 6 (n = 15)	Overall (N = 74)
Number of AEs	448	245	128	79	38	28	105	1071
No AEs	7 (9.5)^b^	7 (11.5)	7 (18.4)	11 (34.4)	7 (36.8)	6 (33.3)	1 (6.7)	1 (1.4)
≥ 1AE	67 (90.5)	54 (88.5)	31 (81.6)	21 (65.6)	12 (63.2)	12 (66.7)	14 (93.3)	73 (98.6)
≥ 1Possibly, probably, or definitely talabostat-related AEs	50 (67.6)	37 (60.7)	14 (36.8)	11 (34.4)	10 (52.6)	10 (55.6)	9 (60.0)	59 (79.7)
≥ 1Grade 3 or 4 AEs	15 (20.3)	11 (18.0)	10 (26.3)	6 (18.8)	4 (21.1)	2 (11.1)	3 (20.0)	42 (56.8)
Discontinued talabostat due to ≥ 1 AE	8 (10.8)	1 (1.6)	0	1 (3.1)	1 (5.3)	0	3 (20.0)	14 (18.9)
≥ 1SAE	9 (12.2)	8 (13.1)	3 (7.9)	2 (6.3)	1 (5.3)	1 (5.6)	0	23 (31.1)
Deaths	10 (13.5)	12 (19.7)	6 (15.8)	5 (15.6)	1 (5.3)	1 (5.6)	4 (26.7)	39 (52.7)

AE = adverse event; SAE = serious adverse event

**a **Safety population, n = 74.

**b **N (%) of patients.

**Table 7:** Adverse events in ≥ 3 patients overall by system organ class and preferred term events.

System Organ Class^a^ Preferred Term	Cycle 1 (n = 74)^b^	Cycle 2 (n = 61)	Cycle 3 (n = 38)	Cycle 4 (n = 32)	Cycle 5 (n = 19)	Cycle 6 (n = 18)	>Cycle 6 (n = 15)	Overall (N = 74)
**Blood and Lymphatic System Disorders**	**24 (32.4)^c^**	**17 (27.9)**	**11 (28.9)**	**9 (28.1)**	**5 (26.3)**	**3 (16.7)**	**4 (26.7)**	**41 (55.4)**
Anaemia^d^	14 (18.9)	9 (14.8)	6 (15.8)	3 (9.4)	1 (5.3)	1 (5.6)	2 (13.3)	29 (39.2)
Thrombocytopenia^e^	8 (10.8)	3 (4.9)	3 (7.9)	6 (18.8)	5 (26.3)	2 (11.1)	4 (26.7)	18 (24.3)
Neutropenia^f^	8 (10.8)	3 (4.9)	6 (15.8)	1 (3.1)	3 (15.8)	0	2 (13.3)	14 (18.9)
Leukopenia^g^	1 (1.4)	2 (3.3)	3 (7.9)	1 (3.1)	1 (5.3)	0	1 (6.7)	7 (9.5)
**Cardiac Disorders**	**2 (2.7)**	**2 (3.3)**	**0**	**0**	**1 (5.3)**	**0**	**0**	**5 (6.8)**
Palpitations	2 (2.7)	0	0	0	1 (5.3)	0	0	3 (4.1)
**Ear and Labyrinth Disorders**	**11 (14.9)**	**4 (6.6)**	**5 (13.2)**	**0**	**1 (5.3)**	**0**	**1 (6.7)**	**19 (25.7)**
Tinnitus	8 (10.8)	3 (4.9)	4 (10.5)	0	0	0	0	13 (17.6)
Hypoacusis	3 (4.1)	2 (3.3)	2 (5.3)	0	0	0	0	5 (6.8)
**Eye Disorders**	**4 (5.4)**	**3 (4.9)**	**5 (13.2)**	**1 (3.1)**	**2 (10.5)**	**0**	**3 (20.0)**	**13 (17.6)**
Vision blurred	0	2 (3.3)	1 (2.6)	1 (3.1)	1 (5.3)	0	1 (6.7)	6 (8.1)

**a **System organ classes are presented alphabetically and preferred terms are listed by decreasing frequency and ordered by the "Overall" column. A patient with multiple occurrences of an AE is counted only once for the AE category. A patient who reported ≥ 2 AEs with different preferred terms within the same system organ class was counted only once in the system organ class total. AEs that do not meet frequency criteria for inclusion in the table are included in the system organ class totals.

**b **Safety population, n = 74.

**c **N (%) of patients.

**d **Includes terms: anaemia, anaemia NOS, red blood cell count decreased, and haemoglobin decreased.

**e **Includes terms: thrombocytopenia and platelet count decreased

**f **Includes terms: neutropenia and neutrophil count decreased.

**g **Includes terms: leukopenia and leukopenia NOS.

Ten patients (10/74 [13.5%]) had AEs which were considered by the investigator as definitely related to talabostat. These AEs included 7 patients with events of edema (including peripheral, localized, facial, and periorbital edema, fluid retention, and edema NOS), two patients with nausea, and one incident each of rigors, performance status decreased, weight increased, myalgia, genital edema, pruritis, or peripheral cyanosis. With the exception of performance status decrease, which occurred during the extended treatment cycles (>cycle 6), these events occurred exclusively in cycles 1 and/or 2. AEs experienced by ≥ 3 patients by dose level of talabostat are presented in Table 8. The percentage of patients experiencing AEs were similar between dose groups in most body system organ classes.

**Table 8:** Adverse events in ≥ 3 patients by talabostat dose level.

	----Talabostat Dose BID---		
System Organ Class^a^	300 mcg	400 mcg	Overall
Preferred term	(n = 74)^b^	(n = 44)	(N = 74)
**Blood and Lymphatic System Disorders**	**32 (43.2)^c^**	**19 (43.2)**	**41 (55.4)**
Anaemia^d^	20 (27.0)	11 (25.0)	29 (39.2)
Thrombocytopenia^e^	13 (17.6)	8 (18.2)	18 (24.3)
Neutropenia^f^	12 (16.2)	5 (11.4)	14 (18.9)
Leukopenia^g^	4 (5.4)	4 (9.1)	7 (9.5)
			
**Cardiac Disorders**	**2 (2.7)**	**3 (6.8)**	**5 (6.8)**
Palpitations	2 (2.7)	1 (2.3)	3 (4.1)
			
**Ear and Labyrinth Disorders**	**15 (20.3)**	**5 (11.4)**	**19 (25.7)**
Tinnitus	10 (13.5)	4 (9.1)	13 (17.6)
Hypoacusis	4 (5.4)	1 (2.3)	5 (6.8)
			
**Eye Disorders**	**9 (12.2)**	**7 (15.9)**	**13 (17.6)**
Vision blurred	2 (2.7)	4 (9.1)	6 (8.1)
			
**Gastrointestinal Disorders**	**54 (73.0)**	**21 (47.7)**	**58 (78.4)**
Nausea	38 (51.4)	11 (25.0)	44 (59.5)
Vomiting NOS	31 (41.9)	10 (22.7)	38 (51.4)
Constipation	22 (29.7)	7 (15.9)	25 (33.8)
Diarrhoea NOS	9 (12.2)	5 (11.4)	13 (17.6)
Abdominal pain NOS	3 (4.1)	4 (9.1)	7 (9.5)
Dyspepsia	2 (2.7)	3 (6.8)	5 (6.8)
Stomatitis	1 (1.4)	2 (4.5)	3 (4.1)

BID = twice daily

**a **System organ classes are presented alphabetically and preferred terms are listed by decreasing frequency and ordered by the "Overall" column. A patient with multiple occurrences of an AE is counted only once for the AE category. A patient who reported ≥ 2 AEs with different preferred terms within the same system organ class was counted only once in the system organ class total. AEs that do not meet frequency criteria for inclusion in the table are included in the system organ class totals.

**b **Safety population, n = 74.

**c **N (%) of patients.

**d **Includes terms: anaemia, anaemia NOS, red blood cell count decreased, and haemoglobin decreased.

**e **Includes terms: thrombocytopenia and platelet count decreased.

**f **Includes terms: neutropenia and neutrophil count decreased.

**g **Includes terms: leukopenia and leukopenia NOS.

Over a third of the patients (27/74 [36.5%]) had AEs considered by the investigator as definitely related to cisplatin. The majority of these events involved the hematopoietic system (15 patients; 11 with neutropenia, febrile neutropenia, and/or neutrophil count decreased) or gastrointestinal disorders (14 patients; 11 with vomiting NOS). Of the 4 most common AEs experienced by patients in this study, the majority were considered possibly, probably, or definitely related to cisplatin (43/44 events of nausea, 35/38 vomiting, 32/37 fatigue, and 27/29 events of anemia). A summary of AEs by severity and by relationship to talabostat or cisplatin is presented in Table 9 by cycle and overall.

**Table 9:** Adverse events by severity and causality.

	Cycle 1 (n = 74)^a^	Cycle 2 (n = 61)	Cycle 3 (n = 38)	Cycle 4 (n = 32)	Cycle 5 (n = 19)	Cycle 6 (n = 18)	>Cycle 6 (n = 15)	Overall (N = 74)
**Severity**								
Grade 1	19 (25.7)^b^	13 (21.3)	8 (21.1)	8 (25.0)	6 (31.6)	8 (44.4)	8 (53.3)	6 (8.1)
Grade 2	33 (44.6)	30 (49.2)	13 (34.2)	7 (21.9)	2 (10.5)	2 (11.1)	3 (20.0)	25 (33.8)
Grade 3	10 (13.5)	9 (14.8)	8 (21.1)	4 (12.5)	2 (10.5)	2 (11.1)	2 (13.3)	28 (37.8)
Grade 4	3 (4.1)	0	1 (2.6)	1 (3.1)	2 (10.5)	0	1 (6.7)	8 (10.8)
Grade 5	2 (2.7)	2 (3.3)	1 (2.6)	1 (3.1)	0	0	0	6 (8.1)
**Relationship to talabostat^c^**								
Not related	6 (8.1)	11 (18.0)	7 (18.4)	5 (15.6)	0	1 (5.6)	3 (20.0)	6 (8.1)
Unlikely	11 (14.9)	6 (9.8)	10 (26.3)	5 (15.6)	2 (10.5)	1 (5.6)	2 (13.3)	8 (10.8)
Possible	33 (44.6)	31 (50.8)	9 (23.7)	10 (31.3)	8 (42.1)	7 (38.9)	4 (26.7)	38 (51.4)
Probable	10 (13.5)	2 (3.3)	4 (10.5)	0	1 (5.3)	2 (11.1)	3 (20.0)	11 (14.9)
Definite	7 (9.5)	4 (6.6)	1 (2.6)	1 (3.1)	1 (5.3)	1 (5.6)	2 (13.3)	10 (13.5)
**Relationship to cisplatin^c^**								
Not related	3 (4.1)	5 (8.2)	5 (13.2)	3 (9.4)	1 (5.3)	2 (11.1)	4 (26.7)	3 (4.1)
Unlikely	4 (5.4)	5 (8.2)	5 (13.2)	2 (6.3)	3 (15.8)	2 (11.1)	2 (13.3)	2 (2.7)
Possible	14 (18.9)	11 (18.0)	4 (10.5)	4 (12.5)	1 (5.3)	2 (11.1)	1 (6.7)	11 (14.9)
Probable	28 (37.8)	18 (29.5)	9 (23.7)	6 (18.8)	5 (26.3)	5 (27.8)	5 (33.3)	30 (40.5)
Definite	18 (24.3)	15 (24.6)	8 (21.1)	6 (18.8)	2 (10.5)	1 (5.6)	2 (13.3)	27 (36.5)

**Note: **A patient who reported ≥ 2 adverse evens (AEs) with the same preferred term was counted only once for that term using the most severe incidence. A patient who reported ≥ 2 AEs with different preferred terms within the same system organ class was counted only once in the system organ class total using the most severe incidence. A patient who reported ≥ 2 AEs with different same system organ class was counted only once in the total using the most severe incidence.

**a **Safety population, n = 74.

**b **N (%) of patients.

**c **As determined by the investigator.

The number and percent of patients with AEs were also analyzed by the initial cisplatin dose the patient received (100 mg/m^2 ^or 75 mg/m^2^). Patients whose initial cisplatin dose was 75 mg/m^2 ^experienced fewer AEs in the hematopoietic system (18/39 [46.2%]) as well as in the organ system category of AE's (13/39 [33.3%]), compared to those who received the 100 mg/m^2 ^dose (23/35 [65.7%] and 19/35 [54.3%], respectively).

Twenty-three patients (23/74 [31.1%]) reported SAEs; 15 of these patients were receiving talabostat at 300 mcg BID at the time of their event and 8 had dose-escalated to 400 mcg BID. The incidence of SAEs was relatively constant over the 6 cycles of treatment, ranging from 5.3 to 13.1% of patients in a given cycle experiencing an SAE. There were no SAEs reported in the extended treatment phase of the study (> Cycle 6).

Eleven SAEs were reported in more than one patient: dehydration was reported in 6 patients; vomiting, dizziness, acute renal failure, dyspnea, and hypotension NOS were each reported in 3 patients; and 2 patients each experienced nausea, fatigue, pain NOS, renal failure NOS, or orthostatic hypotension. One patient experienced SAEs of peripheral edema, facial edema, and peripheral cyanosis that were considered definitely related to talabostat. A second patient experienced SAEs of anasarca, dehydration, and renal failure NOS, all considered probably related to talabostat. Both patients recovered from the events.

The incidence of specific hematologic events was analyzed (Table 6). Few patients experienced AEs of grade 4 neutropenia, febrile neutropenia, grade 3 or grade 4 anemia, or infections requiring hospitalization. Grade 3/4 neutropenia and thrombocytopenia was observed in 18.9% and 24.3% of patients, respectively. No trends for an effect of talabostat on these hematologic parameters were evident in this study.

Overall, there were a total of 39 (39/74 [52.7%]) deaths in this study, the majority of which (36/39) were due to PD. Eleven patients died within 30 days of receiving talabostat: 5 patients died due to PD, melanoma was the cause of death for 3 additional patients, one patient had a myocardial infarction, and 2 patients died of organ failure (end organ failure and renal failure). Hepatic/renal failure also contributed to the death of one patient with metastatic melanoma. The death from renal failure was considered possibly related to both talabostat and cisplatin, and the myocardial infarction was considered unlikely related to talabostat but possibly related to cisplatin. The other deaths were considered not related or unlikely to be related to the study medications.

Eighteen patients (18/74 [24.3%]) experienced ≥ 1 AE that led to discontinuation of study medication. Fourteen of these patients (14/74 [18.9%]) discontinued talabostat (6 patients discontinued talabostat only and 8 patients discontinued both talabostat and cisplatin); 4 patients discontinued cisplatin, but continued receiving talabostat.

## Discussion

Metastatic melanoma carries a grave prognosis, with overall survival of less than 12 months despite significant efforts to develop novel therapeutic agents. Talabostat is an orally available dipeptidyl peptidase inhibitor with immunogenic properties that has demonstrated therapeutic effects in a mouse model of melanoma [28].

The results from this phase II trial of talabostat and cisplatin in metastatic melanoma failed to show significant improvement over currently available treatments. Of 74 patients in the ITT population, those who received a single dose of talabostat, six objective responses, all PRs, were recorded for a response rate of 8.1% (6/74). Five of these responses were also in the evaluable population (those who did not progress within the first 21 days of treatment), yielding a response rate of 12.5% (5/40) for this population. The duration of the response ranged from 62 to 287 days. By the end of treatment or extended treatment, however, most evaluable patients were reported to have PD.

The estimate of median PFS using the Kaplan-Meier survival analysis algorithm for the ITT population was 92.0 days, based on the investigator assessment using RECIST criteria. The estimates of PFS appeared greater for patients with an M1b classification and escalated dose of talabostat. The estimate of median overall survival was 230.0 days, regardless of whether patients starting a new anti-melanoma treatment were censored. Overall survival estimates were greater for patients who dose-escalated and for patients with no prior chemotherapy.

In this population of patients with advanced disease, 56/74 (75.7%) patients were unable to complete 6 cycles (18 weeks) of talabostat, primarily due to PD. There were 15 patients who completed 6 cycles of talabostat and continued to receive additional cycles of talabostat.

Overall, the combination of talabostat and cisplatin was well tolerated compared to historical data using cisplatin alone. The most frequent AEs were nausea, vomiting, fatigue, anemia, edema, and constipation. Most of these events were considered related to cisplatin. The majority of patients experienced either a grade 2 or a grade 3 AE as their most severe event. The percentages of patients experiencing AEs and the types of AEs were similar between the groups who did and did not dose-escalate. This safety profile is consistent with phase 1 studies conducted in 120 healthy male volunteers, in which talabostat was well tolerated at single daily doses up to 2400 μg and when administered as a single dose for seven days at doses up to 1800 μg [30]. However, given the toxicity profile that we observed to cisplatin and a slightly higher early death rate than expected related to progressive disease, further safety assessment is necessary.

## Conclusion

In conclusion, despite promising preclinical evidence, combination treatment with talabostat and cisplatin did not significantly affect disease progression in this study. Antitumor activity was observed in 5/40 (12.5%) of evaluable patients, suggesting the possibility of clinical benefit in a subset of melanoma patients. Based on its low toxicity profile, additional research may be warranted to investigate the potentiating antitumor effects of talabostat on other chemotherapeutic and biologic agents, perhaps in patients with earlier stage disease.

It is unclear if the antitumor activity of talabostat as seen in pre-clinical models involves the induction of immunologically active cytokines and chemokines thereby mediating an immune response or inhibition of tissue remodeling via FAP. No clinical assessment of talabostat in FAP-expressive tumors has as yet been performed. Although clinical development of talabostat has focused on the immune-mediated activity, further assessment of talabostat in patients with FAP-expressive cancers may be worthwhile.

## Competing interests

The authors declare that they have no competing interests.

## Authors' contributions

JN, BJ and MU established the protocol design. CCC, NS, JS, SPA, SJO, GF, ACP and JN were clinical investigators who carried out the study. BJ carried out the immunoassays and the molecular genetic studies. RME and JM drafted the manuscript. All authors read and approved the final manuscript.

## Pre-publication history

The pre-publication history for this paper can be accessed here:

http://www.biomedcentral.com/1471-2407/9/263/prepub
